# Current concepts of the crosstalk between lncRNA and E2F1: shedding light on the cancer therapy

**DOI:** 10.3389/fphar.2024.1432490

**Published:** 2024-07-25

**Authors:** Peng Huang, Feng Wen, Qiu Li

**Affiliations:** ^1^ Division of Abdominal Tumor Multimodality Treatment, Cancer Center, West China Hospital, Sichuan University, Chengdu, Sichuan, China; ^2^ Department of Medical Oncology, Cancer Center, West China Hospital, Sichuan University, Chengdu, Sichuan, China; ^3^ Department of Radiation Oncology, Cancer Center, West China Hospital, Sichuan University, Chengdu, Sichuan, China

**Keywords:** lncRNA, E2F1, cancer progression, immune response, gene therapy

## Abstract

Long noncoding RNAs (lncRNAs) constitute a distinctive subset of RNA molecules with limited protein-coding potential, which exert crucial impacts on various biological activities. In the context of cancer, dysregulated lncRNAs function as essential regulators that affect tumor initiation and malignant progression. These lncRNAs serve as competitive endogenous RNAs (ceRNAs) through sponging microRNAs and regulating the expression of targeted genes. Moreover, they also directly bind to RNA-binding proteins, which can be integrated into a complex mechanistic network. E2F1, an extensively studied transcription factor, mediates multiple malignant behaviors by regulating cell cycle progression, tumor metastasis, and therapeutic response. Emerging evidence suggests that lncRNAs play a pivotal role in regulating the E2F1 pathway. This review aims to elucidate the intricate gene regulatory programs between lncRNAs and E2F1 in cancer progression. We elaborate on distinct mechanistic networks involved in cancer progression, emphasizing the potential of the lncRNAs/E2F1 axes as promising targets for cancer therapy. Additionally, we provide novel perspectives on current evidence, limitations, and future directions for targeting lncRNAs in human cancers. Fully deciphering the intricate network of lncRNA/E2F1-mediated regulatory mechanisms in cancer could facilitate the translation of current findings into clinical course, such efforts ultimately significantly improve the clinical prognosis of cancer patients.

## 1 Introduction

Cancer is a major global health concern, posing significant life-threatening risks in both developed and developing countries (Zhou et al., 2024). Despite advancements in improving clinical prognosis in the past few decades, cancer still presents an immense clinical challenge due to its high mortality rates, posing heavy burdens for public health and global medical expenses (Torre et al., 2016; Cao et al., 2024). The crucial treatment modalities for cancer patients including surgical resection, systematic chemotherapy, radiotherapy, immunotherapy, and molecular targeted therapy, which are associated with systemic toxicity and therapeutic resistance, impeding therapeutic efficacies and their clinical application (Khan et al., 2024; Murthy and Are, 2024). Therefore, developing feasible strategies for cancer is essential, but the intricate pathological mechanisms underlying cancer progression pose a formidable challenge, underscored by multi-faceted considerations of therapeutic efficacies and adverse events (Doostmohammadi et al., 2024; Galluzzi et al., 2024).

Cancer is perceived as a malignant disease characterized by various genetic dysregulations and epigenetic irregularities, leading to elevated expression of oncogenes, which mechanistically promote carcinogenesis by multiple molecular mechanisms including activating the mRNA transcription and augmenting the downstream protein translation (Gong and Li, 2024; Ju et al., 2024). However, protein-coding genes only account for 2%, even though approximately 85% of the human genes are transcribed (Sherman and Salzberg, 2020). Emerging evidence has uncovered that these non-coding genes participate in transcribing a group of noncoding RNAs (ncRNAs) that exert an essential role in the epigenetic modulation of downstream genes (Xu et al., 2023; Yang et al., 2023). They can be classified into two groups based on size: small ncRNAs, such as microRNAs (miRNAs), which are approximately 22 nucleotides in length, and long non-coding RNAs (lncRNAs), which are longer than 200 nucleotides with limited coding potential (Thapa et al., 2024). Numerous studies have indicated a crucial role for lncRNAs in modulating gene expression at transcriptional and translational levels (Siddique et al., 2024). A growing interest in the realm of carcinogenesis has demonstrated that lncRNAs drive tumor initiation and prompt malignant progression by regulating chromatin structure, mRNA stabilization, and protein translation, and acting as competitive endogenous RNAs (ceRNAs) for influencing target gene expression (Huang P. et al., 2024; Saranya et al., 2024; Song et al., 2024; Xiang et al., 2024).

Transcription factors (TFs) are acknowledged as a collection of regulatory proteins that exert significant impacts on gene expression and protein synthesis by binding to certain DNA sequences (Bushweller, 2019; Kant et al., 2022). These changes result in a variety of outcomes such as tissue maintenance, inflammation regulation, and determining cell phenotype (Kant et al., 2022; Li et al., 2022). In the setting of malignant disease, dysregulated TFs are pivotal in the survival and proliferation of tumor cells, and are evident across numerous cancer types (Ahmad et al., 2023). Existing experimental studies have associated functional alterations and expression patterns of TFs with cancer initiation and progression, including SOX2 (Novak et al., 2020), NF-κB (Yu H. et al., 2020), c-Myc (Gao et al., 2023), Snail (Kielbik et al., 2023), signal transducer and activator of transcription (STAT) (Li YJ. et al., 2023), and E2F (Kent and Leone, 2019), which are considered essential TFs influencing cellular homeostasis.

**FIGURE 1:**
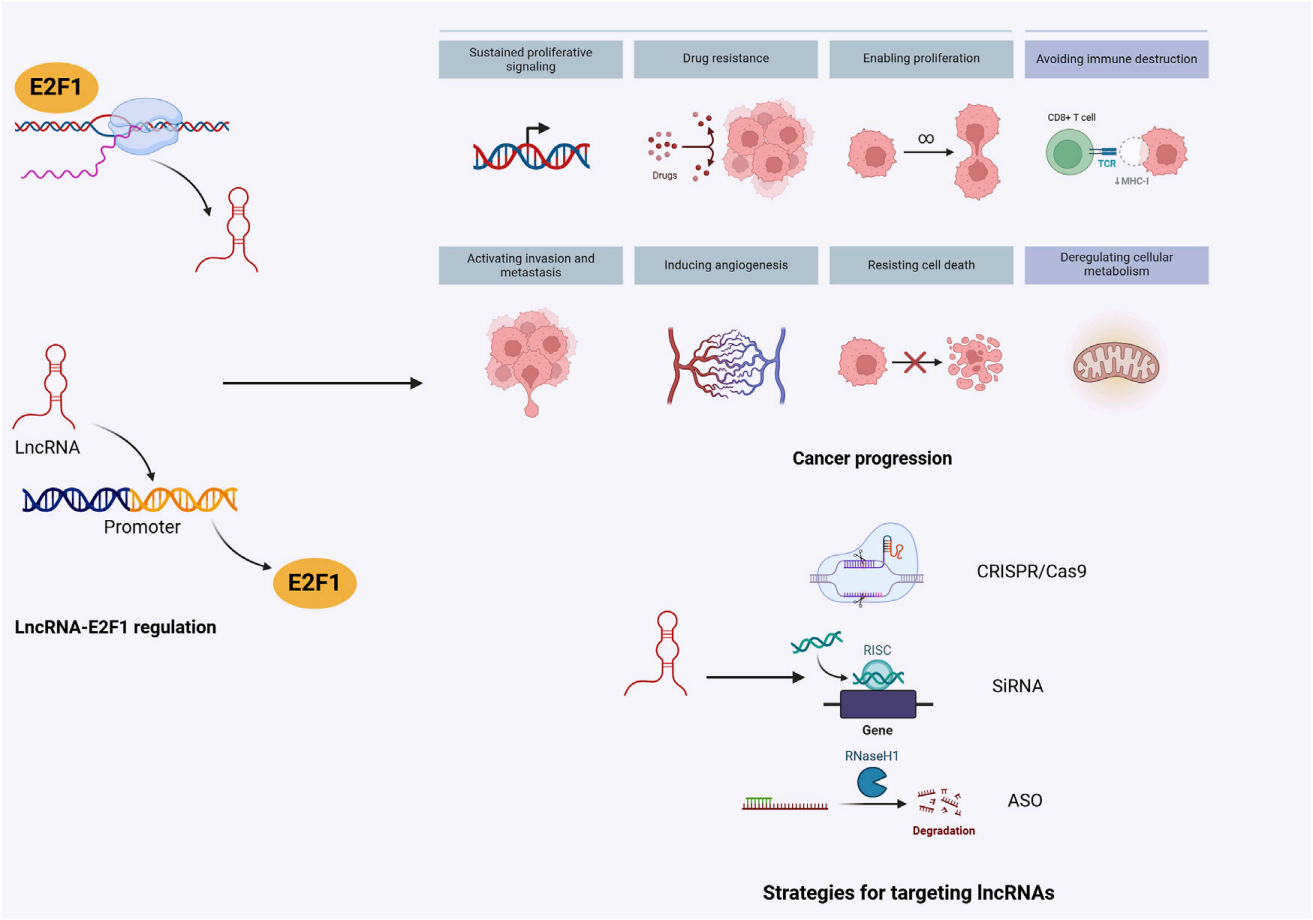
The concise overview of lncRNA/E2F1 crosstalk in cancer.

Regarding E2Fs specifically, there have identified eight members, out of which E2F1 has been extensively studied (Fang et al., 2020a; Chun et al., 2020; Kassab et al., 2023). E2F1 significantly contributes to cell cycle regulation and apoptosis induction in human cancer cells (Meng and Ghosh, 2014). Moreover, there is ample evidence uncovering a direct regulation of E2F1 by lncRNAs, which further promotes malignant behaviors including cell proliferation, metastasis, stemness, and therapeutic response (Priyanka et al., 2022; Barczak et al., 2023; Yu et al., 2023). Notably, dysregulation of the lncRNA-E2F1 axis can be affected by certain signaling pathways, which provide novel insights into drug development for cancer therapy (Logotheti et al., 2020; Xiao et al., 2022). In this review, we emphasize current evidence of lncRNA-E2F1 interaction in the context of malignancies and highlight the feasible preclinical therapeutic modalities for targeting lncRNAs or E2F1, aiming to facilitate the translation of molecular crosstalk into clinical application, which holds immense promises for tumor eradication and ameliorating prognosis (Figure 1).

## 2 Deregulation of E2F1 in cancer progression

The E2F1 gene is situated within the human chromosome 20 region 11 zone 2 (20 q11.2), spanning approximately 11 kilobases in length, which encodes the E2F1 protein that plays a crucial role in various biological processes (Fouad et al., 2020). E2F1 predominantly performs its biological roles through enhancing the expression of numerous downstream genes related to cell cycle regulation, DNA damage response, cellular self-renewal, and tissue development (Ertosun et al., 2016; Denechaud et al., 2017; Manickavinayaham et al., 2020).

E2F1 is deregulated in the setting of human cancers including breast, colorectal, lung, prostate, ovarian, and bladder cancer (Chun et al., 2020; Logotheti et al., 2020; Jing et al., 2022; Priyanka et al., 2022; Yu et al., 2023). Existing evidence has indicated that E2F1 was an essential predictor of prognosis in cancer patients. E2F1 was highly expressed in esophageal squamous cell carcinoma (ESCC) cases and associated lymph node metastasis (Mega et al., 2005). Furthermore, overexpression of E2F1 significantly shortened overall survival (OS) in lung cancer patients observed over 200 months, while this trend was also noted in hepatocellular carcinoma (HCC) patients (Gao et al., 2016; Liu Y. et al., 2022). Indeed, E2F1 plays a critical role in cancer progression. For example, E2F1 expression was upregulated by non-SMC Condensin II Complex Subunit D3 (NCAPD3) in colorectal cancer (CRC) cells, which in turn promoted the transcription of pyruvate dehydrogenase kinase 1 (PDK1) and PDK3 genes, significantly enhancing Warburg effect and leading to advanced disease (Jing et al., 2022). E2F1 directly stimulated protease, serine S1 family member 22 (PRSS22) transcription, which facilitated efficient cleavage of Annexin A1 (ANXA1), producing an N-terminal peptide to activate formyl-peptide receptor-2 (FPR2)/ERK cascade, thus favoring the invasive phenotype in breast cancer (Song et al., 2022). In line with this, Zafar et al. reported that E2F1 enhanced the expression of kinesin superfamily 26A (KIF26A), abolishing p21 expression, consequently favoring cell cycle progression, resulting in tumor growth in the mouse model of breast cancer (Xu J. et al., 2020). In lung cancer, elevated E2F1 was associated with invasion and metastasis of cancer cells (Wang T. et al., 2017; Zhang and Shi, 2023). Mechanistically, E2F1 dampened Zinc finger E-box binding homeobox 2 (ZEB2) expression and promoted the epithelial-mesenchymal transformation (EMT) (Wang T. et al., 2017), which revealed the potential for suppressing distant metastasis by targeting E2F1.

In addition to influencing malignant transformation, E2F1 significantly regulates therapeutic response to cancer cells. E2F1 was reported to orchestrate ABCG2 expression and drive chemotherapeutic drug efflux by targeting ABCG2, which could be restored by inhibiting ABCG2 (Rosenfeldt et al., 2014). Moreover, E2F1/insulin-like growth factor receptor (IGF-1R) axis mediated the activation of phosphoinositide 3-kinase (PI3K)/AKT pathway, promoting BRAF inhibitor resistance in melanoma (Liu et al., 2020). These findings uncover novel pathways in multidrug resistance and reveal previously unrecognized functions of E2F1 that are pertinent to cancer therapy.

## 3 Deregulated lncRNAs in cancer progression

**FIGURE 2:**
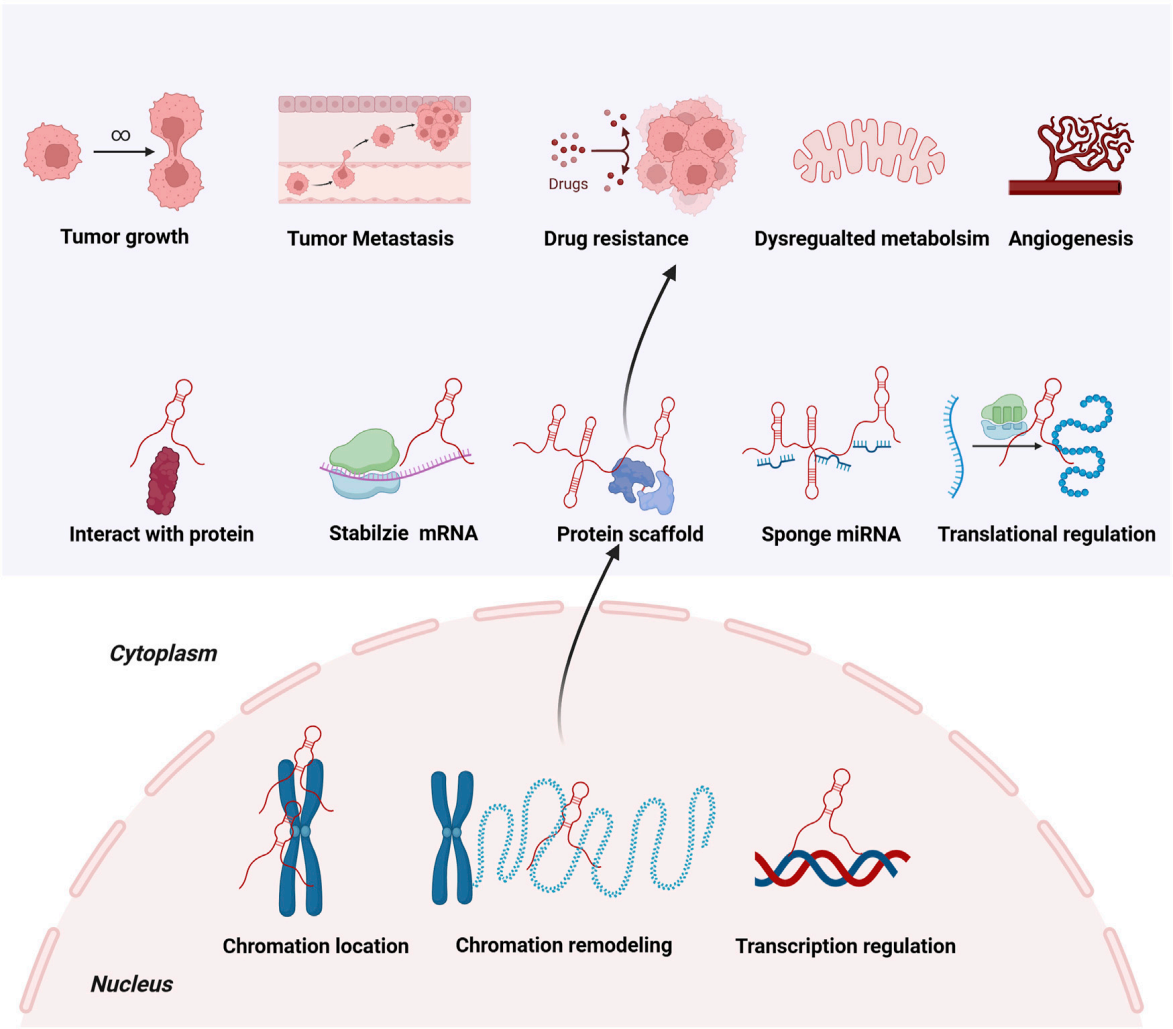
Molecular mechanisms of lncRNAs in the regulation of cancer progression.

A myriad of well-defined lncRNAs have undergone extensive research, leading to the elucidation of their functional mechanisms. Within the nucleus, lncRNAs modulate gene transcription and mediate epigenetic alterations via interactions with chromatin and remodeling chromatin (Bridges et al., 2021). In the cytoplasm, lncRNAs contribute to signaling transduction, mRNA stabilization, and translational regulation (Bridges et al., 2021). Notably, some lncRNAs act as ceRNAs, sequestering miRNAs to modulate the activities and abundance of RNA-binding proteins, thereby influencing post-translational modifications (Yang et al., 2023). Moreover, lncRNAs serve as scaffolds to assemble intricate protein networks involved in tightly modulated signaling pathways (Schmitz et al., 2016). Through diverse mechanisms, lncRNAs exert crucial roles in numerous biological processes (Figure 2).

An emerging focus of lncRNA research revolves around gaining deeper insights into the molecular networks of tumorigenesis (Sakurai and Ito, 2024; Thapa et al., 2024). Recognizing the pivotal control of lncRNAs on various facets of malignant transformation including cell proliferation, growth, metastasis, metabolism, and drug resistance, the interest in elucidating its modulation is significantly reasoned (Hashemi et al., 2022; Wang S. et al., 2023). Metastasis-associated lung adenocarcinoma transcript-1 (MALAT-1) is deregulated in various human cancers (Yan H. et al., 2023; Li GZ. et al., 2023). MALAT1 was found to sponge miR-1-3p, upregulating B-related factor 2 expression, which in turn promoted cell viability and growth in HCC (Li GZ. et al., 2023). In multiple myeloma (MM), MALAT1 acted as ceRNA of miR-15a and miR-16 to upregulate the expression of vascular endothelial growth factor A (VEGFA), which significantly facilitated angiogenesis and led to tumor growth in a MM mouse model (Yan H. et al., 2023). Min et al. (2024) reported that lncRNA MIR100HG exhibited elevated expression levels in lung cancer cells. Further mechanistic studies have disclosed that MIR100HG directly targeted the miRNA-5590-3p/DCBLD2 cascade, which facilitated cell proliferation, invasion, and migration (Min et al., 2024). In contrast with this, lncRNA TINCR was downregulated in pancreatic cancer tissues and dampened EMT by suppressing the Wnt/β-catenin signaling pathway (Wei and Zhu, 2024), which serves as an essential activator of tumor metastasis (Zhan et al., 2017). Moreover, Liu and colleagues reported that CCAT5 was transactivated by the Wnt/β-catenin/transcription factor 3 (TCF3) cascade in gastric cancer cells (Liu et al., 2024). Mechanistic studies have uncovered that CCAT5 directly disturbed the Src homology 2 domain-containing protein tyrosine phosphatase 1 (SHP-1)-mediated dephosphorylation process of STAT3, resulting in accelerating tumor growth, metastasis and resistance to oxaliplatin (Liu et al., 2024). Linc01056 has been identified as a key regulator of sorafenib resistance in HCC (Chan et al., 2024). Knockdown of linc01056 activated peroxisome proliferator-activated receptor alpha (PPARα), leading to elevated fatty acid metabolism and abrogated glycolysis, while aggravating cell resistance to sorafenib (Chan et al., 2024). These findings uncover that deregulated lncRNAs hold great potential as appealing targets for cancer therapy, which awaits further investigations (Table 1).

**TABLE 1:** Deregulated lncRNAs in cancer progression.

LncRNA	LncRNA expression	Cancer	Mechanism	Function	References
MALAT1	Up	HCC	Sponged miR-1-3p, upregulating B-related factor 2 expression	Promoted cell viability and growth	Li et al. (2023b)
MALAT1	Up	Multiple myeloma	Acted as a ceRNA of miR-15a and miR-16 to upregulate the expression of VEGFA	Facilitated angiogenesis and tumor growth	Yan et al. (2023a)
MIR100HG	Up	Lung cancer	MIR100HG targeted the miRNA-5590-3p/DCBLD2 cascade	Facilitated cell proliferation, invasion, and migration	Min et al. (2024)
TINCR	Down	Pancreatic cancer	Suppressed the Wnt/β-catenin signaling pathway	Inhibited EMT	Wei and Zhu (2024)
CCAT5	Up	Gastric cancer	CCAT5 was transactivated by the Wnt/β-catenin/TCF3 cascade	Accelerated tumor growth, metastasis and resistance to oxaliplatin	Liu et al. (2024)
Linc01056	Down	HCC	Knockdown of linc01056 activated PPARα, leading to elevated fatty acid metabolism and abrogated glycolysis	resensitizing cancer cell to sorafenib	Chan et al. (2024)

## 4 The lncRNA and E2F1 regulatory networks

Emerging evidence has revealed that lncRNAs and E2F1 appear to establish a feedback loop because lncRNA could encourage E2F1 transcription, which in turn upregulates lncRNA expression. For example, lncRNA SNHG15 was upregulated in gastric cancer (GC) and E2F1 could interact with the promoter of SNHG15, leading to an increase in its expression (Duan et al., 2024). Concurrently, SNHG15 sponged miR-24-3p to upregulate the mRNA expression of E2F1, which suppressed ferroptosis and promoted cancer progression (Duan et al., 2024). These findings presented a promising therapeutic avenue for GC treatment. DANCR was another lncRNA that upregulated the expression of E2F1 (Yan et al., 2024). Mechanistically, DANCR sequestering miR-34c-5p in breast cancer cells and enhanced cell proliferation and migration (Yan et al., 2024). Additionally, E2F1 enhanced DANCR transcription by directly binding to its promoter in breast cancer cells (Yan et al., 2024). Based on these facts, blocking E2F1-lncRNA interaction may provide a potential therapeutic approach for tackling cancer.

## 5 LncRNAs-E2F1 crosstalk in cancer progression

Evidence continually emerges supporting the link between lncRNAs and E2F1 in regulating tumorigenesis in different cancers. Although previous sections have touched upon the involvement of lncRNAs in cancer progression, we will delve into current investigations demonstrating the molecular actions of these lncRNAs regulated by specifically TF, namely, E2F1 in different cancers. Additionally, evidence supporting lncRNA-mediated E2F1 modulation will also be expounded (Table 2).

**TABLE 2:** The molecular mechanisms of lncRNA/E2F1 crosstalk in human cancers.

LncRNA	LncRNA expression	Upstream regulator	Cancer	Mechanism	Function	References
MNX1-AS1	Up	MNX1-AS1	NSCLC	MNX1-AS1 directly triggers the phase separation of IGF2BP1, maintaining E2F1 and c-Myc mRNA stability	Promotes cell proliferation and tumor growth	Zhu et al. (2022)
TAZ-AS202	Up	TAZ-AS202	NSCLC	TAZ-AS202 upregulates E2F1 expression, enhancing EPHB2 and activating EPH-Ephrin pathway	Promotes tumor growth and metastasis	Gobbi et al. (2023)
HIT	Up	HIT	NSCLC	Upregulates E2F1 expression	Promotes cancer porgression	Park et al. (2018)
EPEL	Up	EPEL	NSCLC	Upregulates E2F1 expression	Promotes cancer porgression	Yu et al. (2017)
SVIL-AS1	Down	SVIL-AS1	LUAD	SVIL-AS1 is induced by METTL3, which facilitates E2F1 degradation	Promotes cell apoptosis	Hu et al. (2022)
GAS5	Down	GAS5	NSCLC	GAS5 inhibits E2F1 expression	Promotes cell apoptosis	Shi et al. (2015)
GAS6-AS1	Down	GAS6-AS1	NSCLC	Suppresses GLUT1 expression by interacting with E2F1	Inhibits glycolysis and tumor growth	Luo et al. (2021)
SBF2-AS1	Up	SBF2-AS1	LUAD	Sponges miR-338-3p and miR-362-3p to upregulate E2F1 expression	Promotes cancer progression	Chen et al. (2019)
PTTG3P	Up	E2F1	NSCLC	E2F1 transcriptionally activates PTTG3P, which interactes with ILF3 to stabilize MAP2K6 mRNA.	Promotes cancer progression	Wang et al. (2023b)
SNHG3	Up	E2F1	NSCLC	E2F1 induces SNHG3 expression, thus activating TGF-β and IL-6/JAK2/STAT3 pathways	Promotes tumor EMT and metastasis	Shi et al. (2020)
SBF2-AS1	Up	E2F1	NSCLC	E2F1 induces SBF2-AS1 expression, which upregulates GRB2 through sequestering miR-362-3p	Promotes tumor metastasis	Wang and Wang (2020)
MCF2L-AS1	up	E2F1	NSCLC	E2F1 induces MCF2L-AS1 expression, which binds to ELAVL1 and stabilizes CCND1 mRNA	Promotes gefitinib resistance	Shan et al. (2022)
FTH1P3	Up	E2F1	NSCLC	Recruits LSD1 and epigenetically suppresses TIMP3 expression	Promotes gefitinib resistance	Zheng et al. (2020)
LINC00847	Up	E2F1	NSCLC	LINC00847 is transcriptionally activated by E2F1 and modulates miR-147a/IFITM1 axis	Promotes cancer progression	Li et al. (2021a)
LINC00662	Up	LINC00662	NSCLC	Sponges miR-320d to enhance E2F1 expression	Promotes cancer progression	Lv et al. (2021)
LINC00511	Up	LINC00662	Breast cancer	LINC00511 sponges miR-185-3p, recovering E2F1 expression and promoting Nanog transcription	Aggravates the sphere-formation ability and stemness of cancer	Lu et al. (2018)
MALAT1	UP	MALAT1	Breast cancer	Targets miR-124/CDK4/E2F1 signaling pathway	Promotes cancer progression	Feng et al. (2016)
DANCR	UP	E2F1	Breast cancer	E2F1-responsive DANCR Sequesteres miR-34c-5p to aggravate E2F1 transcription	Promotes cancer progression	Yan et al. (2024)
RP11-19E11.1	Up	E2F1	Breast cancer	E2F1 promotes RP11-19E11.1 transcription	Promotes enzastaurin resistance	Giro-Perafita et al. (2020)
AGPG	Up	AGPG	Breast cancer	AGPG physically activates E2F1 via interacting with PURα and blocking the binding of E2F1 to PURα	Promotes tumor growth and endocrine resistance	Yu et al. (2023)
ERINA	Up	ERINA	Breast cancer	ERINA is directly transactivated by the ER binding site and interfers with the interaction between E2F1 and RB1	Promotes tamoxifen and palbociclib resistance	Fang et al. (2020b)
CDKN2B-AS1	Up	CDKN2B-AS1	HCC	CDKN2B-AS1 interacts with E2F1 and exacerbates the transcription of GNAZ	Promotes cancer progression	Tao et al. (2023)
LINC01089	Up	E2F1	HCC	E2F1-induced LINC01089 binds to hnRNPM, which undermines N6-methyladenosine-mediated DIAPH3 mRNA stability and elicits ERK/Snail signaling pathway	Promotes EMT and metastasis	Su et al. (2023)
LINC01004	Up	E2F1	HCC	E2F1 activates the transcription of LINC01004	Promotes EMT and metastasis	Li et al. (2023c)
KDM4A-AS1	Up	E2F1	HCC	Elevated KDM4A-AS1 expression mediated by E2F1 stabilizes AURKA via recruiting ILF3, provoking PI3K/AKT pathway	Promotes cancer progression	Shen et al. (2023)
lnc-APUE	Up	lnc-APUE	HCC	lnc-APUE sequesteres miR-20b and ameliorates the repression of E2F1 expression	Promotes cancer progression	Li et al. (2021b)
SNHG6	Up	SNHG6	HCC	Sponges miR-204-5p to upregulate E2F1	Promotes cancer progression	Chen et al. (2021)
LINC00852	Up	LINC00852	HCC	Sponges miR-625 to upregulate E2F1	Promotes cancer progression	Chen (2022)
ZEB1-AS1	Up	ZEB1-AS1	HCC	Sponges miR-299-3p to upregulate E2F1	Promotes cancer progression	Mu et al. (2021)
DUBR	Up	DUBR	HCC	SP1-transcribed lncRNA DUBR recruited E2F1 and enhanced CIP2A expression, whereas DUBR also sequesteres miR-520d-5p to enhance the stability of E2F1 mRNA and elicits Notch1 signaling pathway	Promotes cancer stemness and oxaliplatin resistance	Liu et al. (2022b)
H19	Up	H19	CRC	H19 activates E2F1 signaling pathway	Promotes cell proliferation and tumor growth	Ohtsuka et al. (2016)
NEAT1	Up	NEAT1	CRC	NEAT1 abrogates KDM5A expression via interacting with E2F1, which suppressed the KDM5A/H3K4me3-mediated repression of Cul4A expression, resulting in activated Wnt signaling pathway	Promotes cancer progression	Shen et al. (2021)
LINC01703	Up	LINC01703	CRC	LINC01703 promotes PI3K/AKT signaling pathway via miR-205-5p/E2F1 axis	Promotes cell proliferation and migration	Xu et al. (2024)
CRNDE	Up	CRNDE	CRC	CRNDE sponges miR-136 and indirectly targets E2F1	Promotes cancer metastasis and oxaliplatin resistance	Gao et al. (2017)
DLGAP1-AS2	Up	DLGAP1-AS2	rectal cancer	DLGAP1-AS2 enhances the radiotherapy resistance of rectal cancer cells by interacting with E2F1 to increase CD151 expression through activating the AKT/mTOR/cyclinD1 signaling pathway	Promotes radiotherapy resistance	Xiao et al. (2022)
EGFR-AS1	Up	EGFR-AS1	Gastric cancer	EGFR-AS1 promotes TUBA1C expression, which aggravates E2F1 expression and facilitates the G1 phase to the S phase transition	Promotes tumor growth and metastasis	Wang et al. (2023c)
NNT-AS1	Up	NNT-AS1	Gastric cancer	NNT-AS1 sponges miR-424 and elevates E2F1 expression	Promotes cancer progression	Chen et al. (2018)
GAS5	Down	GAS5	Gastric cancer	GAS5 disturbs E2F1 expression, and modulates miR-34c expression, which leads to the repressed p38MAPK signaling pathway	Inhibits cancer progression	Sun et al. (2014), Gupta et al. (2022)
PIN1P1	Up	PIN1P1	Gastric cancer	CREB1 activates PIN1P1 transcription, which upregulates YBX1 expression, resulting in enhanced E2F1 and PIN1 expression	Promotes cancer progression	Wang et al. (2024)
HOXA11-AS	Up	E2F1	Gastric cancer	E2F1 mediates the transcription of HOXA11-AS, which establishes a mechanistic model comprising the crosstalk between E2F1/HOXA11-AS/miR-1297/EZH2 axis and LSD1/HOXA11-AS/EZH2 complex	Promotes cancer progression	Sun et al. (2016)
LSINCT5	Up	E2F1	Gastric cancer	E2F1 induces LSINCT5 expression	Promotes cell proliferation, growth, and EMT	Niu et al. (2020)
HCG18	Up	E2F1	Gastric cancer	E2F1 induces HCG18 expression	Promotes cell proliferation, growth, and EMT	Qi et al. (2018)
SLC16A1-AS1	Up	SLC16A1-AS1	Bladder cancer	SLC16A1-AS1 interacts with E2F1 to enhance the expression of SLC16A1/MCT1 and PPARA.	Promotes aerobic glycolysis and exacerbates fatty acid β-oxidation, favoring an invasive phenotype	Logotheti et al. (2020)
TMPO-AS1	Up	E2F1	Bladder cancer	E2F1-induced TMPO-AS1 fuels the binding of E2F1 to OTUB1, enhances the deubiquitination and stability of E2F1, which unveils a novel TMPO-AS1/E2F1/OTUB1 regulatory program	Promotes cell proliferation and aggressiveness	Zhang et al. (2021)
GAS5	Down	GAS5	Prostate cancer	GAS5 facilitates the transcription of P27^Kip1^ by recruiting E2F1 to its promoter	Inhibits cell proliferation and tumor growth	Luo et al. (2017)

### 5.1 Lung cancer

GLOBOCAN 2023 data indicates that lung cancer contributes to the most cancer-related fatalities globally, and this trend is consistent across all ethnic groups, posing a significant public health challenge worldwide (LoPiccolo et al., 2024). Lung cancer cases are categorized into non-small cell lung cancer (NSCLC) and small-cell lung cancer (SCLC), out of which NSCLC accounts for approximately 85% (LoPiccolo et al., 2024). Although great advancements have been made in early diagnosis and systemic therapy, the 5-year survival rates of lung cancer patients remain dismal, emphasizing the urgent requirement for novel therapeutic modalities (LoPiccolo et al., 2024).

By overlapping the differentially expressed lncRNAs from the GENCODE database and NSCLC-related microarray, which contented 42 paired NSCLC and normal lung tissues, Zhu and his colleagues found that lncRNA MNX1-AS1 was upregulated and associated with advanced tumor stage (Zhu et al., 2022). Elevated MNX1-AS1 directly triggered the phase separation of insulin-like growth factor 2 mRNA-binding protein 1 (IGF2BP1), maintaining E2F1 and c-Myc mRNA stability by binding to their untranslated regions, which remarkably increased c-Myc and E2F1 protein levels (Zhu et al., 2022). The MNX1-AS1/IGF2BP1/c-Myc/E2F1 cascade activated cell-cycle regulatory signals to facilitate cell colony formation and tumor growth both *in vitro* and *in vivo*, providing fresh perspectives on the control of c-Myc and E2F1 signaling in the malignant transformation of NSCLC, indicating that the MNX1-AS1/IGF2BP1 axis could potentially function as a promising target for NSCLC therapy (Zhu et al., 2022). In line with this, lncRNA TAZ-AS202 was identified as a TAZ-transcribed RNA molecule that supported the activation of EPH-Ephrin signaling pathways (Gobbi et al., 2023). Further investigations demonstrated that dysregulated TAZ-AS202 upregulated E2F1 expression, which accelerated NSCLC growth and metastasis by transcriptionally activating a myriad of oncogenes including Eph receptor B2 (EPHB2), a key component of EPH-Ephrin pathway (Gobbi et al., 2023). Similarly, several studies have identified that lncRNA HIT and EPEL directly targeted E2F1, prompting cell proliferation, invasion, and migration in NSCLC models (Yu et al., 2017; Park et al., 2018). LncRNA SVIL antisense RNA 1 (SVIL-AS1) was decreased in lung adenocarcinoma (LUAD) cells, abolished cell-cycle progression, and restrained cell proliferation (Hu et al., 2022). Mechanistically, SVIL-AS1 could be induced by methyltransferase-like 3 (METTL3), which facilitated E2F1 ubiquitination and proteasomal degradation, which could be reversed by E2F1 overexpression (Hu et al., 2022). GAS5 was another lncRNA that was downregulated in NSCLC and promoted cell apoptosis by interacting with E2F1 (Shi et al., 2015). In LUAD, GAS6-AS1 was downregulated and suppressed the expression of glucose transporter protein-1 (GLUT1), a pivotal regulator of glycolysis. Overexpression of GLUT1 reversed the inhibitory impact of GAS6-AS1 on the aggressive nature and the metabolic reprogramming. Subsequent explorations revealed that GAS6-AS1 directly bound to E2F1, thereby abolishing GUT1 transcription (Luo et al., 2021). Moreover, lncRNA SBF2-AS1 formed a novel gene regulatory model in the early-stage LUAD by sponging miR-338-3p and miR-362-3p to regulate E2F1 expression, which might function as a promising diagnostic marker (Chen et al., 2019).

E2F1 could act as the upstream regulator of lncRNAs by inducing their transcription. For example, lncRNA PTTG3P interacted with interleukin enhancer-binding factor 3 (ILF3) to stabilize the mRNAs of MAP2K6 and E2F1, which induced malignant transformation in the mouse model of NSCLC (Wang J. et al., 2023). Interestingly, increased E2F1 in turn transcriptionally activated PTTG3P, establishing a reciprocal regulatory loop (Wang J. et al., 2023). Moreover, E2F1-induced lncRNA SNHG3 enhanced NSCLC cell proliferation, invasion, and EMT by activating transforming growth factor-β (TGF-β) and IL-6/JAK2/STAT3 signaling pathway (Shi et al., 2020). SBF2-AS1 was associated with distant metastasis of NSCLC patients (Chen et al., 2020; Wang and Wang, 2020). E2F1 contributed to the overexpression of SBF2-AS1, which led to the upregulated expression of GRB2 through sequestering miR-362-3p, ultimately facilitating tumor metastasis (Wang and Wang, 2020). Gefitinib, the orally administered tyrosine kinase inhibitor for lung cancer patients harboring the epidermal growth factor receptor (EGFR) mutations across therapy lines (Wu and Shih, 2018). Acquired resistance to gefitinib significantly impedes its therapeutic efficiencies, with a myriad of studies exploring its potential mechanisms (Wu and Shih, 2018). Shan et al. (2022) reported that the upregulation of MCF2L-AS1 induced by E2F1 directly bound to ELAVL1, which stabilized Cyclin D1 (CCND1) mRNA, inducing cell resistance to gefitinib. Notably, ferritin heavy chain 1 pseudogene 3 (FTH1P3) was also induced by E2F1 and facilitated gefitinib resistance through recruiting lysine-specific demethylase 1 (LSD1) and epigenetically suppressing the tissue inhibitors of metalloproteinases 3 (TIMP3) (Zheng et al., 2020). These findings indicate that targeting E2F1-lncRNA interactions and their downstream molecules might be feasible strategies for overcoming gefitinib resistance, which provides fresh insights into the treatment of advanced NSCLC patients.

CeRNA is an emerging regulatory mechanism focusing on the crosstalk among lncRNA, miRNA, and target genes (Chen et al., 2020; Li H. et al., 2021). Exosomal lncRNA LINC00662 functioned as a molecular sponge for miR-320d and indirectly regulated E2F1 expression, thus accelerating cell proliferation, metastasis, and repressing cell cycle arrest of NSCLC *in vitro* (Lv et al., 2021). Notably, LINC00847 was transcriptionally activated by E2F1 and contributed to NSCLC progression through targeting modulating miR-147a/IFITM1 axis (Li H. et al., 2021).

**FIGURE 3:**
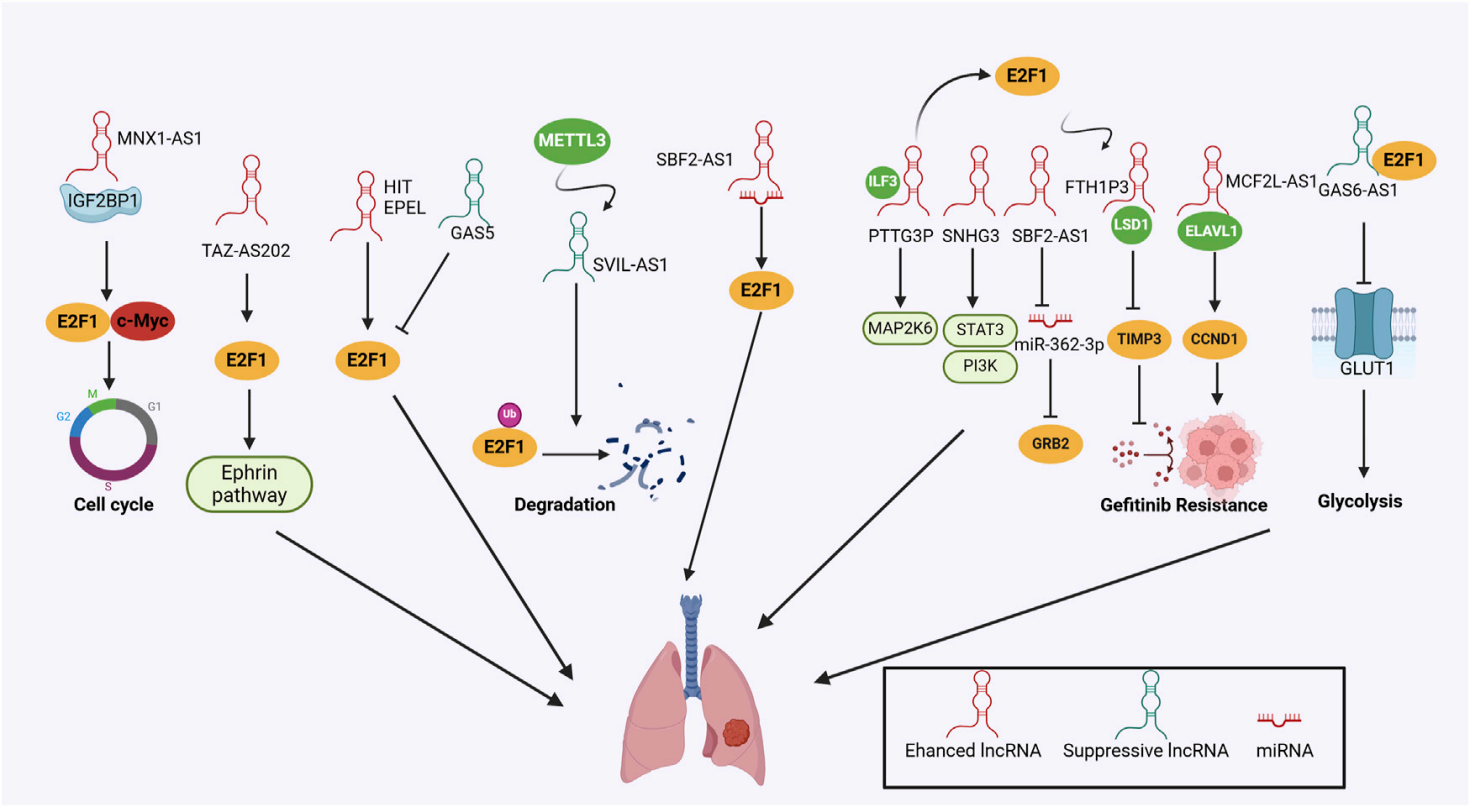
The mechanisms of the lncRNA/E2F1 crosstalk in lung cancer.

As research technologies advance, there is immense potential to delve into the relationship between E2F1 and lncRNAs in lung cancer, leading to a more detailed understanding of lncRNA mechanisms. Interfering with the E2F1-lncRNA network could emerge as a pivotal approach for precise lung cancer therapy (Figure 3).

### 5.2 Breast cancer

Breast cancer stands out as the most common malignancy among women, featuring highly heterogeneous and various intrinsic molecular subtypes, harboring distinct therapeutic responses (Xu X. et al., 2020). Recent studies have considered lncRNAs as key regulators in breast cancer progression. For example, overexpressed LINC00511 aggravated the sphere-formation ability and stemness of breast cancer. Mechanically, LINC00511 acted as a ceRNA for miR-185-3p, thereby recovering E2F1 expression and promoting the transcription of Nanog, an essential stemness factor (Lu et al., 2018). MALAT1 played an essential role in breast cancer development by targeting miR-124/CDK4/E2F1 signaling pathway (Feng et al., 2016). DANCR sequestered miR-34c-5p to aggravate E2F1 transcription, leading to enhanced malignant behaviors (Yan et al., 2024). Furthermore, E2F1 also activated DANCR transcription, forming an E2F1/DANCR/miR-34c-5p positive feedback loop (Yan et al., 2024), presenting novel approaches for tackling cancer progression.

**FIGURE 4:**
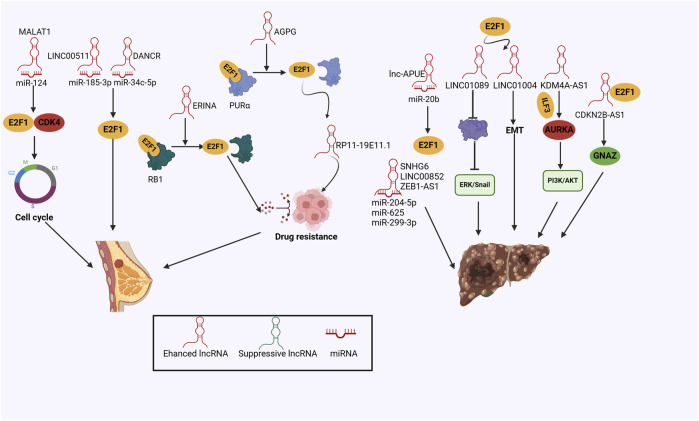
The mechanisms of the lncRNA/E2F1 crosstalk in breast cancer and hepatocellular carcinoma.

Ensuring optimal therapeutic efficacy while minimizing adverse effects is essential for effective breast cancer treatment, aiming to maintain a high quality of life for patients (Xu X. et al., 2020). Drug resistance still presents a remarkable challenge for breast cancer, contributing to tumor recurrence and metastasis (Herzog and Fuqua, 2022). By using RNA-sequencing data derived from the TCGA database, Giro-Perafita, and his colleagues focused on RP11-19E11.1, a chromatin-associated lncRNA targeted by E2F1, which was essential for cell survival, cell cycle progression and associated with decreased sensitivity to enzastaurin of breast cancer cells (Giro-Perafita et al., 2020). AGPG was measured to be upregulated in endocrine-resistant breast cancer specimens and related to poor prognosis in ERα+ breast cancer patients (Yu et al., 2023). Mechanistically, AGPG physically activated E2F1 via interacting with PURα and blocking the binding of E2F1 to PURα, while AGPG inhibition significantly repressed tumor growth in the endocrine-resistant mouse model (Yu et al., 2023). Notably, nuclear lncRNA ERINA was directly transactivated by the ER binding site and aggravated cell cycle progression through interfering with the interaction between E2F1 and RB1, which remarkably promoted tamoxifen and palbociclib resistance, demonstrating that targeting lncRNAs holds great potential for combating endocrine-resistant tumors (Fang et al., 2020b) (Figure 4).

### 5.3 Hepatocellular carcinoma

HCC represents a significant public health challenge globally, being the most prevalent type of primary liver cancer (Vogel et al., 2022). Studies have shown that epigenetic changes are closely associated with HCC progression (Ganesan and Kulik, 2023). In recent years, researchers have taken an interest in exploring the functional role of lncRNAs in hepatocarcinogenesis (Ganesan and Kulik, 2023). Tao et al. (2023) revealed that upregulation of lncRNA CDKN2B-AS1 was observed in HCC samples. CDKN2B-AS1 exerted a pro-oncogenic role by interacting with E2F1 and exacerbating the transcription of G protein subunit alpha Z (GNAZ), thereby aggravating cell proliferation, and growth, and repressing cell apoptosis. LINC01089 was identified as a super-enhancer lncRNA that was induced by E2F1 (Su et al., 2023). Highly expressed LINC01089 acted as a promoter of HCC cell EMT and metastasis through binding to heterogeneous nuclear ribonucleoprotein M (hnRNPM), which undermined N6-methyladenosine (m6A)-mediated DIAPH3 mRNA stability and elicited ERK/Snail signaling pathway (Su et al., 2023). LINC01004 was another SE-lncRNA transcribed by E2F1, significantly exacerbating cell growth and metastasis, providing mechanistic perspectives on hepatocarcinogenesis and the HCC treatment (Li J. et al., 2023). Elevated KDM4A-AS1 expression mediated by E2F1 significantly stabilized Aurora kinase A (AURKA) mRNA via recruiting interleukin enhancer-binding factor 3 (ILF3), which provoked PI3K/AKT pathway and augmented tumorigenicity in HCC experimental models (Shen et al., 2023).

A bioinformatics study performed by Dong and Zhan (2022) has annotated several distinct lncRNA/miRNA/E2F1 regulatory loops in the tumor formation of HCC, although lacking experimental evidence. Li S. Y et al. (2021) annotated the oncogenic role of lncRNA accelerating proliferation by upregulating E2F1 (lnc-APUE) by loss-of-function analyses. Mechanistic evidence disclosed that lnc-APUE sequestered miR-20b and ameliorated the repression of E2F1 expression, facilitating cell cycle progression and cell proliferation. However, the upregulation of lnc-APUE could be antagonized by hepatocyte nuclear factor 4 alpha (HNF4α), while its downregulation supported HCC development (Li SY. et al., 2021). Additionally, SNHG6, LINC00852, and ZEB1-AS1 worked as the sponges for miR-204-5p, miR-625, and miR-299-3p respectively, relieving the suppression of E2F1 expression, leading to exacerbated G1-S phase transition, tumor growth and metastasis (Chen et al., 2021; Mu et al., 2021; Chen, 2022). These findings identified several distinct regulatory axes and represented appealing candidates for novel drug development of HCC.

Oxaliplatin is commonly utilized as a therapeutic option for advanced HCC, but chemotherapy resistance worsens clinical prognosis and provokes tumor recurrence (Liu S. et al., 2022). Current theoretical investigations have identified a novel SP1/DUBR/E2F1-miR-520d-5p/CIP2A regulatory axis and deciphered its molecular actions, that was, SP1-transcribed lncRNA DUBR recruited E2F1 and enhanced CIP2A expression in HCC, whereas DUBR also sequestered miR-520d-5p to stabilize CIP2A protein, which in turn enhanced the stability of E2F1 mRNA and elicited Notch1 signaling pathway, thus accelerating cancer stemness and promoted HCC cell resistance to oxaliplatin (Liu S. et al., 2022), supporting a novel molecular candidate for therapeutic intervention against oxaliplatin resistance and awaiting further verifications in the clinical setting (Figure 4).

### 5.4 Colorectal cancer

**FIGURE 5:**
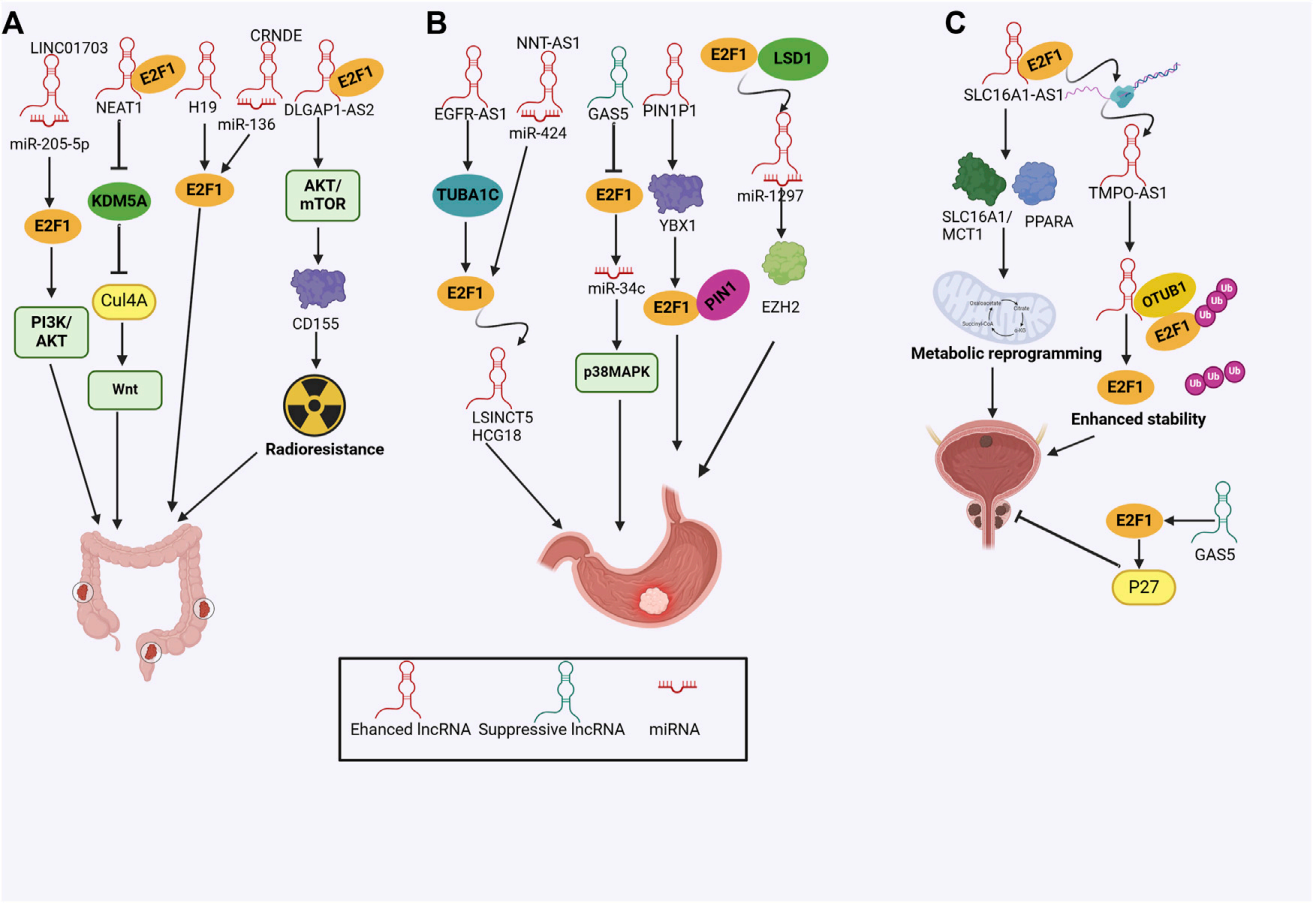
**(A)** The mechanisms of the lncRNA/E2F1 crosstalk in colorectal cancer; **(B)** The mechanisms of the lncRNA/E2F1 crosstalk in gastric cancer; **(C)** The mechanisms of the lncRNA/E2F1 crosstalk in prostate and bladder cancer.

CRC is the third most commonly diagnosed cancer and ranks second in terms of mortality globally (Jiang et al., 2024). Previous studies have reported that lncRNA H19 was involved in CRC tumorigenicity, whereas Ohtsuka et al. underscored the H19-E2F1 cascade in CRC, that was, H19 activated E2F1 signaling pathway to promote cell proliferation and tumor growth (Ohtsuka et al., 2016). In line with this, NEAT1 was found to abrogate KDM5A expression via interacting with E2F1, which suppressed the KDM5A/H3K4me3-mediated repression of Cul4A expression, resulting in activated Wnt signaling pathway and augmented tumor formation (Shen et al., 2021). Notably, the PI3K/AKT pathway could also be regulated in CRC cell proliferation and migration by lncRNA LINC01703 via the miR-205-5p/E2F1 axis (Xu et al., 2024). CRNDE was recently recognized as a key regulator in the metastasis and oxaliplatin resistance of CRC by working as a ceRNA for miR-136 and indirectly targeting E2F1 (Gao et al., 2017). Radioresistance stands as a primary contributor to the failure of cancer treatment (Xiao et al., 2022). LncRNA DLGAP1-AS2 enhanced the radiotherapy resistance of rectal cancer cells by interacting with E2F1 to increase CD151 expression through activating the AKT/mTOR/cyclinD1 signaling pathway (Xiao et al., 2022). These findings deciphered the molecular mechanisms of lncRNA-E2F1 interplay in CRC and emphasized the clinical prospects of targeting lncRNAs or E2F1 (Figure 5A).

### 5.5 Gastric cancer

GC is perceived as one of the most common gastrointestinal malignancies globally and poses a high burden on public health owing to hight morbidity and lethality (Smyth et al., 2020). By global transcriptomic analyses, Sun et al. (2023) reported that E2F1 was the essential TF in GC progression by interacting with a plethora of dysregulated lncRNAs and forming specific regulatory axes. Consistent evidence has also been annotated in the case of lncRNA EGFR-AS1/TUBA1C/E2F1 (Wang H. et al., 2023). Elevated EGFR-AS1 promoted GC growth and metastasis by targeting TUBA1C, which aggravated E2F1 expression and facilitated the G1 phase to the S phase transition (Wang H. et al., 2023). Moreover, lncRNA might sponge miRNA to rescue the E2F1 inhibition, thus promoting disease development. E2F1 functioned as the downstream target of the NNT-AS1/miR-424 axis, leading to enhanced tumorigenesis (Chen et al., 2018). GAS5 was perceived as a tumor suppressor that has been dysregulated in multiple cancers, including GC. In the preclinical evidence, GAS5 could directly disturb E2F1 expression, suppressing GC cell proliferation and growth (Sun et al., 2014). The GAS5-E2F1 interaction also modulated miR-34c expression, which led to the repressed p38MAPK signaling pathway (Gupta et al., 2022), fully elaborating on the molecular crosstalk of GAS5 in GC. In another mechanistic model, the CAMP response element binding protein (CREB1) activated lncRNA PIN1P1 transcription, which upregulated YBX1 expression, resulting in enhanced E2F1 and PIN1 expression, ultimately boosting GC tumorigenicity (Wang et al., 2024).

In the realm of GC models, E2F1 mediated the transcription of HOXA11-AS, which severed as a molecular scaffold by recruiting LSD1 and EZH2 (Sun et al., 2016). Additionally, HOXA11-AS also sponged miR-1297 to antagonize its inhibitory effects on the protein translation process of EZH2, establishing a mechanistic model comprising the crosstalk between E2F1/HOXA11-AS/miR-1297/EZH2 axis and LSD1/HOXA11-AS/EZH2 complex, which supported that HOXA11-AS and E2F1 severed as engaging therapeutic targets against the aggressive growth of GC (Sun et al., 2016). Similarly, E2F1 induced lncRNA LSINCT5 and HCG18 transcriptional activity, leading to increased expression of lncRNAs, which stimulated proliferation, growth, and EMT in GC (Qi et al., 2018; Niu et al., 2020) (Figure 5B).

### 5.6 Urinary tract tumors

As one of the most prevalent urinary tract tumors, the role of lncRNA in bladder cancer has not been fully deciphered. Logotheti and colleagues presented the holistic evidence that lncRNA SLC16A1-AS1 interacted with E2F1 to form an RNA-protein complex, enhancing the expression of SLC16A1/MCT1 and PPARA, which accelerated aerobic glycolysis and exacerbating fatty acid β-oxidation, favoring an invasive phenotype (Logotheti et al., 2020). Through functional assays, Zhang et al. (2021) demonstrated that TMPO-AS1 fueled cell proliferation and aggressiveness of bladder cancer. Mechanistic evidence underscored the upregulation of TMPO-AS1 was attributed to the activity of E2F1. Additionally, TMPO-AS1 fueled the binding of E2F1 to OTU domain-containing ubiquitin aldehyde binding 1 (OTUB1), enhancing the deubiquitination and stability of E2F1, which unveiled a novel TMPO-AS1/E2F1/OTUB1 regulatory program (Zhang et al., 2021). In prostate cancer, GAS5 facilitated the transcription of P27^Kip1^ by recruiting E2F1 to its promoter, repressing cell proliferation and growth (Luo et al., 2017) (Figure 5C).

## 6 E2F1-related lncRNAs in cancer immunotherapy

LncRNA genes represent a diverse group of genes with heterogeneous expression patterns, particularly evident in the context of cancer (Song et al., 2024). LncRNAs, previously thought to be non-coding, may indeed have the capability to encode biological peptides that are shorter than 100 amino acids, which are involved in the modulation of multiple malignant tumor phenotypes (Wu et al., 2020; Zhang et al., 2023). Additionally, lncRNA-derived peptides have been found to regulate immune response. It is worth noting that lncRNA-derived peptides are self-antigens, which should be obliterated during the development of organisms (Reinhard et al., 2020). Intriguingly, the immunogenic peptides showed a tendency towards upregulation in normal thymocytes, indicating that these peptides may evade central tolerance mechanisms in the thymus, such as clonal negative selection, owing to their low expression patterns, and instead, they might be affected by peripheral tolerance mechanisms (Reinhard et al., 2020). Cumulative evidence has reported immunization protocols that delivered lncRNA-derived peptides as a cancer vaccine into cancer models, generating a remarkable immune response against tumor progression (Zhang et al., 2023). By RNA sequencing analysis, Wojciech et al. unveiled that protein arginine methyltransferase (PRMT) 5 and E2F1 exerted striking effects on the expression of various human lncRNA genes including MALAT1, DANCR, TTC28-AS1, LINC00963, and LNCOC1 (Barczak et al., 2023). Notably, E2F1 was highly presented at the promoters of numerous lncRNA genes encoding peptides; approximately 44% of lncRNAs seemed to be targets of E2F1 (Barczak et al., 2023). These lncRNAs produced small peptides that interacted with ribosomes and underwent translation into polypeptides, which were subsequently processed, likely resulting in the generation of peptides that bound to the MHC class I complex (Barczak et al., 2023). Then, they designed two cancer vaccines (ChAdOx1-PepLnc and MVA-PepLnc) that were delivered to the immune system in mice either by loading onto dendritic cells *ex vivo* or by expression from a viral vector, which elicited a robust production of interferon (IFN) γ, reflecting the activated immune response of CD8 T lymphocytes along with significantly hindered tumor growth (Barczak et al., 2023). These findings underscore that E2F1-responsive lncRNAs-derived immunogenic peptides can bind to MHC class I complex and be engineered as cancer vaccines that trigger the CD8 T cell-mediated adaptive immune response, providing remarkably therapeutic benefits.

Programmed cell death ligand 1 (PD-L1) functions as a pivotal immune checkpoint commonly expressed in multiple types of human cancers, exerting a crucial role in immune evasion by repressing activated T lymphocytes via binding to PD-1 (Yi et al., 2022). Understanding the molecular mechanisms governing PD-L1 expression significantly promotes comprehension of the functions of the tumor immunosuppressive microenvironment and augmenting antitumor immune responses (Yi et al., 2022). In the experimental investigation performed by Lin et al. (2023) lncRNA HIF-1α inhibitor at translation level (HITT) was induced by E2F1 upon IFN-γ stimulation. HITT cooperated with the regulator of G protein signaling 2 (RGS2) to bind to the promoter of PD-L1, leading to decreased PD-L1 translation, which augmented T lymphocyte-mediated cytotoxicity and significantly impeded tumor growth in mouse models, emphasizing the activation of HITT as a promising therapeutic avenue to boost cancer immunotherapy (Lin et al., 2023). In line with this, Song and his colleagues expanded the regulatory associations of lncRNA/E2F1 in the immune response to HCC. It revealed that lncRNA CASC11 regulated by YY1 recruited EIF4A3 and stabilized E2F1, activating the NF-κB and PI3K/AKT/mTOR signaling pathway, which further enhanced PD-L1 expression, emphasizing that CASC11/E2F1 might promote HCC progression via PD-L1-mediated immune evasion (Song et al., 2020). One recent pan-cancer analysis has identified a novel lncRNA ribonucleotide reductase small subunit M2 (RRM2) that was overexpressed in thirty types of human cancers. Further mechanistic investigations identified that E2F1 regulated RRM2 expression, which impacted resistance to ICIs via the PI3K/AKT pathway (Wu et al., 2022). These studies provide preclinical evidence that targeting lncRNA functions as a potent avenue to enhance the responsiveness of ICIs.

## 7 Targeting lncRNAs as potent strategies for cancer therapy

Given the prominent roles of lncRNAs in driving cancer progression and their impact on the fundamental characteristics of nearly all malignancies, as well as their involvement in the immune response to tumors, multiple therapeutic strategies have been deployed to target lncRNAs and attenuate their oncogenic functions. So far, the disruption of lncRNA networks has been undertaken through several approaches. LncRNAs possess distinct characteristics from mRNAs and proteins, which enhance their potential as therapeutic targets against cancer.

### 7.1 RNA interference

RNA interference (RNAi) has traditionally served as the standard method for functional screening, contributing to the identification of notable cancer-associated lncRNAs such as MALAT1, LINC00152, and DGCR5 (Wang R. et al., 2017; Nötzold et al., 2017; Li Y. et al., 2023). RNAi applications include using chemically synthesized small interfering RNAs (siRNAs) or deploying short hairpin RNAs (shRNAs) delivered by lentiviral vectors (Gagnon et al., 2014; Booth et al., 2023). Numerous siRNAs have been assessed for their efficacy in silencing lncRNAs within cancer cell lines. MALAT1, an oncogenic lncRNA implicated in cancer metastasis (Feng et al., 2016; Li GZ. et al., 2023), was silenced by a specific siRNA, resulting in reduced tumor growth and metastasis in prostate cancer (Wang R. et al., 2017). DANCR plays a crucial role in the development of multiple types of human cancers, while the invasion phenotype and growth of lung tumors were repressed by shRNA treatment (Yu J. E. et al., 2020). Despite acclimating evidence demonstrating the effective silencing of oncogenic lncRNAs, the functions of RNAi in targeting lncRNAs remain a controversial topic. Some researchers have indicated suboptimal performance, particularly for targets enriched in the nucleus, where many lncRNAs reside, may be attributed to diminished RNAi activity (Gagnon et al., 2014). Furthermore, *in vivo* studies of siRNAs have been challenging to conduct, likely due to the limited bioactivity of siRNAs in living organisms and difficulties in drug delivery (Dowdy, 2017).

### 7.2 Antisense oligonucleotides

Antisense oligonucleotides (ASOs) are short, single-stranded oligonucleotides consisting of 12–25 nucleotides, which undergo chemical modifications to degrade target RNAs through the action of ribonuclease H (RNAse H) that is an enzyme found ubiquitously to cleave RNAs (Booth et al., 2023). The subcutaneous administration of ASO targeting ITGB8-AS1 in CRC-bearing mice markedly delayed tumor growth and reduced the number of liver metastases (Lin et al., 2022). Moreover, utilizing ASO against hypoxia-induced lncRNA LUCAT1 promoted cell apoptosis and re-sensitized CRC cells to chemotherapeutic drugs, indicating a novel therapeutic target for chemotherapy-refractory hypoxic tumors (Huan et al., 2020). When deploying ASO treatments against oncogenic lncRNAs, it’s essential to consider the distinct molecular actions inherent to lncRNAs.

Optimal candidates necessitate optimization for both great therapeutic efficiencies and less untoward effects. Due to their high repetitive contents, lncRNAs could be especially susceptible to off-target toxicities, as this enhances the chances of targeting ASOs containing partial complementarity to multiple off-target lncRNAs with the same repeat sequences (Shen et al., 2019). These instances can be prevented through meticulous experimental validation employing multiple, distinct ASO sequences.

Equally daunting is the task of identifying accessible and potent ASO target sites on the desired lncRNA, which may be obscured by secondary structures or molecular binding partners (Doxtader Lacy et al., 2022). Currently, there are limited publicly available methods for ASO prediction, and the majority of studies depend on costly and time-consuming ASO optimization, which entails synthesizing and testing numerous sequences *in vitro* (Ramasamy et al., 2022). Consequently, ASO prediction stands out as a promising area within RNA therapeutics that is highly conducive to innovation.

### 7.3 CRISPR–Cas9 system

Remarkable advancements in gene editing techniques, including CRISPR–Cas9, offer an innovative therapeutic approach against lncRNAs (Xie et al., 2015). The inactivated Cas9 is paired with transcriptional inhibitors and guided RNAs, guiding this fusion protein toward the targets to trigger silencing (Xie et al., 2015). In this regard, CRISPR/Cas9-mediated silencing of LINC00511, significantly enhanced cell apoptosis by downregulating the expression of anti-apoptotic genes in breast cancer (Azadbakht et al., 2022). Under hypoxic conditions, LUCAT1 was induced by HIF1α in glioma stem-like cells. Functionally, LUCAT1 directly targeted HIF1α, facilitating tumor growth, which could be reversed by CRISPR–Cas9-mediated depletion of LUCAT1 (Huang H. et al., 2024).

It is worth noting that the artificial intelligence technique has reached a point where it can provide predictions and identifications of the single guide RNAs (sgRNAs) within the CRISPR-Cas9 platform, which may provide fresh perspectives on anti-cancer drug development and cancer therapy (Chan et al., 2022). Although CRISPR-Cas9-based gene editing exhibits high efficiency, its broad adoption in clinical studies is hindered by the unforeseen off-target effects (Kozovska et al., 2021; Vicente et al., 2021). Feasible approaches aimed at mitigating undesired editing involve the targeted delivery of the CRISPR system and chemical modification of Cas9, which partially prevent the adverse events of the clinical application (Vicente et al., 2021; Chan et al., 2022). More technological innovations are required to balance the potency and safety of this technology, such efforts ultimately effectively treating cancers.

### 7.4 Current clinical course on lncRNA-mediated therapy strategies

Following the approval by the FDA, the first RNA-based drug was Macugen, a siRNA medication authorized for the treatment of macular degeneration (Nagpal et al., 2007; Starita et al., 2007). Following this milestone, different ASO therapies are currently under clinical trials for conditions like cystic fibrosis (Allaire et al., 2023) and retinitis pigmentosa (Schellens et al., 2023). Another type of RNA therapy includes mRNA, which can be utilized to lower protein levels using Cas9 cleavage methods, or to correct protein mutations at the DNA level through base editing (Gillmore et al., 2021).

While several miRNA mimics and anti-miRNA-based RNA therapies are advancing through phases II or III of clinical development (Ma et al., 2023; Traber and Yu, 2023), treatments utilizing lncRNAs have not yet been integrated into clinical practice. However, promising progress in fields such as molecular biology, immunology, pharmacology, and nanotechnology suggests the potential future adoption of these therapies (Winkle et al., 2021; Entezari et al., 2022). Targeting lncRNAs and their associated RNA-binding proteins in medical treatments is a critical goal for the scientific and medical communities, requiring significant efforts to enhance prospects for patients (Yao et al., 2022). Concerns may arise regarding the importance and consequences of these interactions in comparison to protein-protein, mRNA-protein, and miRNA-protein interactions (Shaath et al., 2022).

Developing strategies to shield therapeutic molecules, allowing for immune evasion and effective uptake, could greatly enhance the efficacy and longevity of administered treatments (Sparmann and Vogel, 2023). Cutting-edge methods, such as administering anti-miRNAs through ultrasound and microbubble-targeted systems for cardiovascular conditions, offer promising opportunities for advancing efficient and feasible RNA-based therapeutics (Pham et al., 2020). The specific therapeutic benefits of lncRNAs are still being uncovered, and in-depth research using experimental and machine learning-based approaches to better understand these interactions will pave the way for the utilization of lncRNA-based treatments in the fight against cancer (Mehmandar-Oskuie et al., 2024; Nemeth et al., 2024).

## 8 Current knowledge for targeting E2F1 in cancer therapy

As discussed above, E2F1 impacts malignant behaviors in various types of human cancers, thus it serves as a candidate for anti-cancer treatment. Multiple peptides were deployed to hinder the DNA-binding ability of E2Fs, exhibiting efficacies in curtailing cell proliferation and tumor growth. A small peptide containing seven-amino acid that were identified by Xie et al. (2013). This peptide, when conjugated with penetratin (PEP), elicited cellular uptake and has been demonstrated to bind to the E2F1 promoter to abrogate its transcription and downregulated E2F-responsive enzymes, leading to notable cytotoxicities in cell lines of lung cancer and prostate cancer (Xie et al., 2013; Xie et al., 2014). Additionally, PEP encapsulation within PEGylated liposomes improved its stability *in vivo* in xenograft models, thereby effectively suppressing tumor growth (Xie et al., 2013). Subsequent studies by Shaik and his colleagues confirmed the efficacy of a functional structure, D-Arg penetratin peptide (D-Arg PEP), as a transcriptional inhibitor of E2F1 (Shaik et al., 2018). This novel compound has demonstrated comparable stability and heightened efficacy for eliminating lung cancer and prostate cancer cells, positioning it as a potent candidate for developing targeted therapy against E2F1-induced tumors (Shaik et al., 2018).

Fungi serve as a rich reservoir of pharmaceutical compounds, including those with anti-tumor properties (Han et al., 2022). Among them, the anti-tumor function of Neurospora crassa has been evaluated in breast cancer models (Han et al., 2022). The Neurospora crassa mixture markedly blocked E2F1 transcription and downregulated lncRNA HOTAIR, thus exhibiting the anti-tumor impacts on cancer invasiveness and growth *in vivo*, while it had less effects on normal mammary cells (Han et al., 2022). Notably, Actinidia chinensis Planch Root extracts (acRoots), bioactive components derived from Chinese medicine, have been shown to suppress cancer cell proliferation, invasion, and metastasis (Wang et al., 2020; Gan et al., 2021). In the setting of hypopharyngeal carcinoma, administration of acRoots repressed tumor growth through decreasing E2F1 expression and inhibiting lncRNA MNX1-AS1 activity (Zheng et al., 2022), which holds immense potential for medical interventions treating E2F1 dysregulated cancers, although more holistic evidence should be presented in the clinical settings.

## 9 Challenges in targeting lncRNA in cancer therapy

Cancer stands as the primary cause of mortality worldwide, commonly diagnosed in advanced stages due to mild symptoms and lacking reliable biomarkers (Zhan et al., 2017). Among the extensively studied genes in the realm of carcinogenesis, E2F1 emerges as a crucial regulator, which functions as an oncogenic TF to provoke tumorigenesis by inducing cell cycle progression and inhibiting apoptosis through modulating target genes (Fang et al., 2020a; Chun et al., 2020).

E2F1 represents a central node interconnected with various signaling pathways implicated in human cancers (Fang et al., 2020a). Emerging evidence has demonstrated that lncRNAs contribute to E2F1 pathway regulation and play multifaceted roles in the disease progression in diverse tumor types (Song et al., 2022; Shen et al., 2023; Zhang and Shi, 2023). By influencing cellular processes like cell proliferation and metastasis in cancer, lncRNAs become integral components of the E2F1 regulatory networks. Moreover, the dysregulated lncRNAs hint at their potential applications in cancer therapy.

**FIGURE 6:**
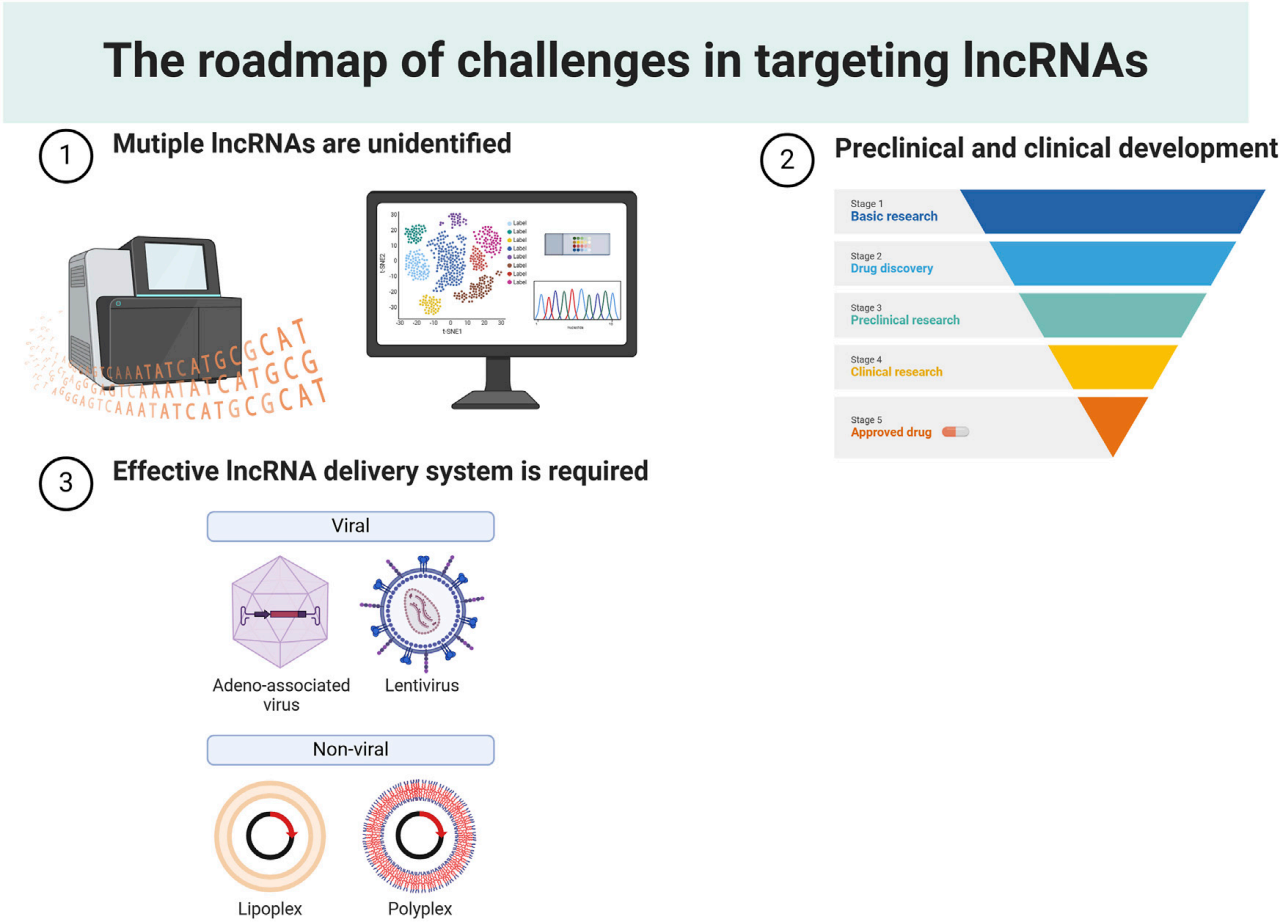
The roadmap of challenges in targeting lncRNAs.

Despite considerable advancements in recent years, lncRNA-based gene therapy still faces several challenges (Figure 6). 1) Numerous unidentified lncRNAs remain to be discovered and characterized. Despite the establishment of several databases for predicting potential functions and investigating mechanistic modes of lncRNAs, the number of lncRNAs cataloged in these databases remains limited (Iwakiri et al., 2016; Yan J. et al., 2023). Additionally, a myriad of lncRNAs newly detected through high-throughput sequencing technology await to be annotated. Consequently, there is a pressing need to develop a high-coverage database and integrate it into emerging sequencing techniques to decipher the role of tumor-specific lncRNAs. 2) The sluggish pace of preclinical and clinical development. LncRNAs are functional molecules with limited evolutionary conservation, which presents a challenge in evaluating off-target toxicities and therapeutic efficacies in experimental models. To address the cost and time delays associated with poor-quality targets, one strategy is to anchor structural conservation for orthologue identification. While these orthologues can not share the same sequences, they demonstrate similarity in their 3D structures (Seemann et al., 2017). Additionally, advancements in human organoids, offer opportunities to boost clinical development in the realm of lncRNA research (Diermeier and Spector, 2017). 3) An effective lncRNA delivery system is required for effective cancer treatment. Researchers have explored various biological materials to facilitate the delivery of lncRNAs. Exosomes, a subset of cell-derived extracellular vesicles (EVs), have shown excellent biocompatibility for cellular uptake (Kalluri and LeBleu, 2020). Their membrane can be engineered, and their small size makes them promising carriers for delivering lncRNAs (Kalluri and LeBleu, 2020). However, the high cost associated with exosome purification and membrane engineering technologies poses challenges for mass production (Liang et al., 2021). Further research into various types of nanoparticles could significantly reduce the costs associated with lncRNA-based drug development. Lipid nanoparticles (LNPs), widely used as gene therapy carriers in clinical trials, have demonstrated success in delivering ASOs for targeting mRNAs (Kon et al., 2023). The emergence of mRNA-contained LNP vaccines during the COVID-19 pandemic underscores the potential advantages of utilizing LNPs for drug delivery (Wilson and Geetha, 2022). Considering the structural similarities between lncRNAs and mRNAs, it is conceivable that lncRNA-contained LNP drugs could become feasible and provide clinical benefits.

While the progression of Andes-1537 into phase 1 clinical trials is promising, the clinical applications of lncRNA-based drugs are largely unexplored, akin to the tip of an iceberg. As a nascent field, lncRNAs hold promising potential in tumor research, offering novel avenues and opportunities for efficiently treating cancers and ultimately eliminating tumors.

## 10 Conclusion

Throughout this review, we have emphasized the critical role of lncRNAs and E2F1 in oncogenesis and the progression of various cancer types by regulating diverse cellular processes and signaling networks. LncRNAs, which have recently been identified as a subset of non-coding RNAs, have attracted considerable attention due to their potential to advance the development of next-generation RNA therapeutics. Their specificity, low toxicity, and ability to interact with other regulatory molecules make them advantageous for targeting complex pathways involved in diseases, particularly cancer. By delving into the interactions between E2F1 and lncRNAs, researchers may uncover the molecular mechanisms underlying malignant transformation and disease advancement, potentially leading to innovative therapeutic approaches for treating cancer. But the potential manipulation of these interactions as a therapeutic strategy for cancer awaits validation through animal models and human clinical trials.
